# Giant metastasis of the thenar eminence revealing ovarian rhabdomyosarcoma

**DOI:** 10.11604/pamj.2020.36.96.23023

**Published:** 2020-06-15

**Authors:** Karima Oualla, Nawfel Mellas

**Affiliations:** 1 Medical Oncology Department, Hassan II University Hospital, Casablanca, Morocco,; 2 Medical Oncology Department, Sidi Mohamed Ben Abdellah University, Fes, Morocco

**Keywords:** Ovary, rhabdomyosarcoma, thenar, metastasis

## Image in medicine

Ovarian rhabdomyosarcoma is extremly rare and the diagnosis might be a real dilemma clinically and histologically. We report here the case of a 19-year-old patient, who presented a progressive increase of the abdominal volume associated with dyspnea and general conditions deterioration. The pelvic MRI showed bilateral ovarian masses associated with peritoneal carcinomatosis. CA125 was elevated (273u/ml) while other markers including bHCG, inhibine, AFP were normal. Simultaneously, the patient developed a cutaneous lesion in the thenar's lodge of the right hand rapidly increasing volume, painful and necrotic. The MRI of the right hand showed a large necrotic tissue mass of the right thenarian lodge without bone involvement which may correspond to a secondary localization. Cutaneous biopsy was performed and revealed a skin localization of undifferentiated malignant tumour and IHC analysis were compatible with alveolar rhabdomyosarcoma. After these findings, an exploratory laparotomy was performed and revealed the presence of two bilateral ovarian masses with ascites of great abundance and large dissemination of carcinomatosis nodules. Several biopsies were performed and the histological analysis with IHC have confirmed the diagnosis of ovarian rhabdomyosarcoma (desmin, myogenin, myoD1 were positive). The patient received chemotherapy based on: vincristine, adriamycin and cyclophosphamide with peritoneal and thenar progression after 6 cycles. Patient refused hand amputation and was proposed for radiotherapy. A second line chemotherapy with etoposide and ifosfamide was established.

**Figure 1:**
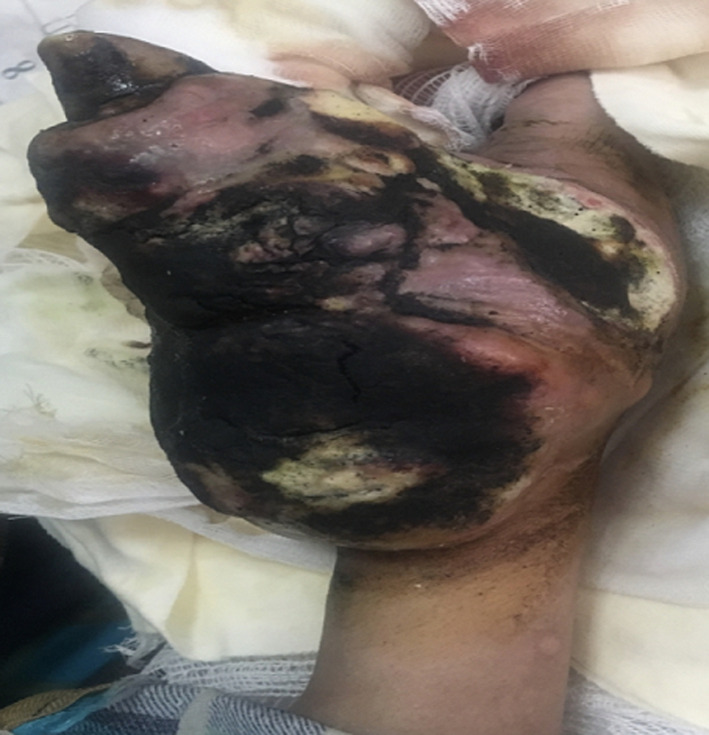
giant metastasis of the thenar eminence revealing ovarian rhabdomyosarcoma

